# Microbiomes of biohydrogen production from dark fermentation of industrial wastes: current trends, advanced tools and future outlook

**DOI:** 10.1186/s40643-022-00504-8

**Published:** 2022-03-05

**Authors:** Eka Latiffah Nadia Dzulkarnain, Jemilatu Omuwa Audu, Wan Rosmiza Zana Wan Dagang, Mohd Firdaus Abdul-Wahab

**Affiliations:** 1 Department of Biosciences, Faculty of Science, Universiti Teknologi Malaysia, 81310 Skudai, Johor Malaysia; 2 Department of Science Laboratory Technology, Modibbo Adama University, PMB 2076, Yola, Adamawa Nigeria; 3 Taiwan-Malaysia Innovation Centre for Clean Water and Sustainable Energy (WISE Centre), Universiti Teknologi Malaysia, 81310 Skudai, Johor Malaysia

**Keywords:** Biohydrogen microbiomes, Biohydrogen production, Dark fermentation, Palm oil mill effluent, Industrial wastes, Molecular biology tools

## Abstract

Biohydrogen production through dark fermentation is very attractive as a solution to help mitigate the effects of climate change, via cleaner bioenergy production. Dark fermentation is a process where organic substrates are converted into bioenergy, driven by a complex community of microorganisms of different functional guilds. Understanding of the microbiomes underpinning the fermentation of organic matter and conversion to hydrogen, and the interactions among various distinct trophic groups during the process, is critical in order to assist in the process optimisations. Research in biohydrogen production via dark fermentation is currently advancing rapidly, and various microbiology and molecular biology tools have been used to investigate the microbiomes. We reviewed here the different systems used and the production capacity, together with the diversity of the microbiomes used in the dark fermentation of industrial wastes, with a special emphasis on palm oil mill effluent (POME). The current challenges associated with biohydrogen production were also included. Then, we summarised and discussed the different molecular biology tools employed to investigate the intricacy of the microbial ecology associated with biohydrogen production. Finally, we included a section on the future outlook of how microbiome-based technologies and knowledge can be used effectively in biohydrogen production systems, in order to maximise the production output.

## Introduction

Dark fermentation is a biological decomposition process reported to be one of the most promising approaches for the treatment of organic wastes. This is also the process commonly used in sustainable bioenergy production. A recent study by the World Bank in 2018 predicted that the global waste production will grow to 3.4 billion tonnes by 2050, with organic wastes generated from agricultural sectors representing more than 50% of the total waste composition (Kaza et al. 2018). This large amount of wastes has to be sustainably managed. For this purpose, dark fermentation can offer two simultaneous benefits of both waste treatment and sustainable bioenergy generation (Wang and Yin 2019). Methane is currently the commonly produced bioenergy from organic wastes, but hydrogen production is also gaining attention, as part of the hydrogen economy, to substitute the hydrogen produced from fossil fuels. Hydrogen has three times higher energy content (120 MJ/kg) than hydrocarbon fuels, and its combustion is clean and carbon free, producing only water as the by-product (Zhang et al. 2020). Dark fermentation is more attractive than the other biological processes, due to the low demand for light (unlike the photosynthetic routes), capable of high biohydrogen production rate, environmentally friendly, versatile substrate utilisation and less energy intensive (Ghimire et al. 2015; Mishra et al. 2019). In addition, the use of organic wastes as feedstocks in dark fermentative biohydrogen production is potentially cost competitive, since organic wastes are relatively abundant, renewable, cheap and highly biodegradable (Sharma et al. 2020).

Various renewable organic wastes such as sake lees, cassava, sago, glycerol, rice straw, vegetable waste, food waste, date seeds, sugarcane molasses, corn stover, alligator weed, oil palm sap and wheat straw have been explored as the potential substrate for dark fermentative biohydrogen production (Chen et al. 2021; Choiron et al. 2020; Li et al. 2020; Liu et al. 2013; Moreno-Andrade et al. 2015; Noparat et al. 2012; Oliveira et al. 2020; Panin et al. 2020; Pason et al. 2020; Rambabu et al. 2020; Saleem et al. 2020; Ulhiza et al. 2018; Zhang et al. 2011). Palm oil mill effluent (POME), a wastewater generated in large quantity during palm oil extraction process is another renewable organic waste of interest that is currently under intense investigations as biohydrogen production substrate (Abdullah et al. 2020; Akhbari et al. 2021; Audu et al. 2021; Jamali et al. 2019; Zainal et al. 2018). The use of both pure, as well as mixed culture as the inoculum in the dark fermentation reactor have been investigated. A mixed culture system is generally more preferable and practical over pure culture system, due to the diverse microbial communities present that can rapidly degrade a wide range of substrates. A strict aseptic condition is also not required, making its handling easier with cheaper cost of operation (Nitipan et al. 2014; Pachapur et al. 2019). Nonetheless, the co-existence of biohydrogen producers with non-biohydrogen producers, and biohydrogen-consumers such as methanogens and homoacetogens in the mixed culture, makes it a very biochemically complex environment. Despite the multiple studies carried out, there is still a gap in the understanding of the biological mechanisms of dark fermentation for biohydrogen production, including the specific microbial community and the trophic interactions (Cabrol et al. 2017; Das 2017). The methane-producing fermentation systems are more well characterised in this aspect.

Microbiomes are classically defined as the community consisting of microorganisms with distinct properties and metabolic functions, interacting with its environment which results in the formation of specific ecological niche (Whipps et al. 1988). The term “microbiome” was often used interchangeably with “microbiota”, but recently there has been efforts to distinguish these two. Berg et al. (2020) defined “microbiota” as the assemblage of living microorganisms (i.e. the bacteria, archaea, fungi, microalgae and the protists, excluding phages, viruses, plasmids, prions, viroids, and free DNA), while the “microbiomes” are the microbiota and their structural elements, metabolites/signal molecules, and the surrounding environmental conditions (Berg et al. 2020). Phages, viruses, plasmids, prions, viroids, and free DNA are part of the microbiomes. This review will refer to this updated definition.

Taxonomic classification of biogas microbiomes is often accomplished using sequence similarity searches against 16S ribosomal RNA (rRNA) gene reference databases, such as SILVA (Akhbari et al. 2021), Greengenes (Oliveira et al. 2020), Ribosomal Database Project (RDP) (Cho et al. 2018) or National Center for Biotechnology Information (NCBI) (Mazareli et al. 2020). However, the genome sequences of biogas-producing microorganisms are underrepresented in these reference databases, which hinder the reliable taxonomic classification for microbiomes present in the biogas production systems (Hassa et al. 2018). Functional roles of biogas microbiome are often inferred to related species in public genome database based on the 16S rRNA gene sequence similarity (Campanaro et al. 2016). Therefore, it is imperative to have a comprehensive reference database to improve the taxonomic annotation of biogas-producing microbiomes across the entire microbial databases. Metagenomics has been used in many biogas-producing studies to decipher the taxonomic diversity, metabolic functions and the physiology of biogas-producing microbiomes. This has led to the compilation of metagenome-assembled genomes (MAGs) belonging to the biogas-producing microbiomes in a repository, called the “Biogasmicrobiome” (https://biogasmicrobiome.env.dtu.dk/) (Campanaro et al. 2020). This database contains a collection of 1600 MAGs of bacterial and archaeal species that underpin various biogas production systems, substantially expanding the existing public genome databases (Campanaro et al. 2020). In addition, Microbial Database for Activated Sludge (MiDAS) Field Guide (https://www.midasfieldguide.org/guide/search) is an ecosystem-specific database for wastewater treatment systems which aims to facilitate collaborative research and compile information on the physiology and ecology of the key microorganisms present in activated sludge wastewater treatment systems (McIlroy et al. 2015). MiDAS 4 offers a comprehensive set of full-length amplicon sequence variant (ASV)-resolved 16S rRNA gene sequences which covers over 31,000 species, allowing researchers to dig into the microbiome compositions of activated sludge, anaerobic digesters and wastewater treatment systems at the genus to species level resolutions (Dueholm et al. 2021).

Dark fermentation for biohydrogen production is mediated by many different groups of microorganisms, to convert complex organic wastes into biohydrogen, volatile fatty acids and carbon dioxide (CO_2_) (Hay et al. 2013). The efficiency and stability of dark fermentation system relies on the syntrophic activity of the microbial community belonging to different functional guilds, working in tight interaction (Cabrol et al. 2017). It has been reported that the understanding of the species composition, specific metabolic functions, and interspecies interactions are often more important than the species richness itself in maintaining the overall performance of the system (Cabrol et al. 2017). The rapid advancement of molecular tools has contributed to the major discoveries of the diversity and structure of the biohydrogen-producing consortia. In a mixed culture system, the microbiomes involved are phylogenetically diverse, with multiple contributions in the biohydrogen production and the breakdown of organic wastes (Cabrol et al. 2017).

This review summarises and evaluates the distinct microbial communities present in a biohydrogen production systems, and the molecular tools that have been used for microbiome analysis in biohydrogen production from industrial wastewater and POME. We also included a future outlook of how microbiome-based technologies and knowledge can be used effectively in biohydrogen production systems, in order to maximise the production output.

## Microbiomes in dark fermentative biohydrogen production

The microorganisms present in dark fermentative biohydrogen production system include both the biohydrogen producers and non-producers. Biohydrogen producers possess the ability to convert complex organic substrates into biohydrogen in the absence of light. They can exist as a single strain or a community of various taxa. They can be found in a diverse environment, such as POME sludge (Jamali et al. 2019; Mahmod et al. 2019; Zainal et al. 2018), sludge from municipal wastewater plants (Chang et al. 2011; Viana et al. 2019), sludge from food waste (Li et al. 2018), cattle dung (Sen and Suttar 2012), pig manure (Wang et al. 2011) and marine sediments (Liu et al. 2018), many of which has been extensively studied. In general, *Clostridium* (Gram positive, spore former) and *Enterobacter* (Gram negative, non-spore former) are the most common biohydrogen-producing genera reported, for mesophilic conditions (Kumar et al. 2018). While under thermophilic and hyperthermophilic conditions, *Clostridium*, *Thermoanaerobacterium*, *Thermotoga* and *Caldicellulosiruptor* dominate (O-Thong 2017). Research on biohydrogen production using the lower temperature-adapted psychrophiles and psychrotrophs are still somewhat limited (Alvarado-Cuevas et al. 2015; Mohammed et al. 2018). In addition, other genera including *Bacillus*, *Ethanoligenens*, *Klebsiella*, *Citrobacter* and *Escherichia* also frequently reported as the biohydrogen producers. Non-biohydrogen producers on the other hand, could interfere with the overall biohydrogen yield, by either consuming the hydrogen produced, competing with the biohydrogen producers for substrates, or inhibit biohydrogen producers with their produced metabolites which eventually decrease the efficiency of the biohydrogen production system as a whole (Cabrol et al. 2017). Inoculum pre-treatment has become necessary in mixed culture systems in order to selectively enrich the biohydrogen producers and inactivate the hydrogen consumers.

**Fig. 1:**
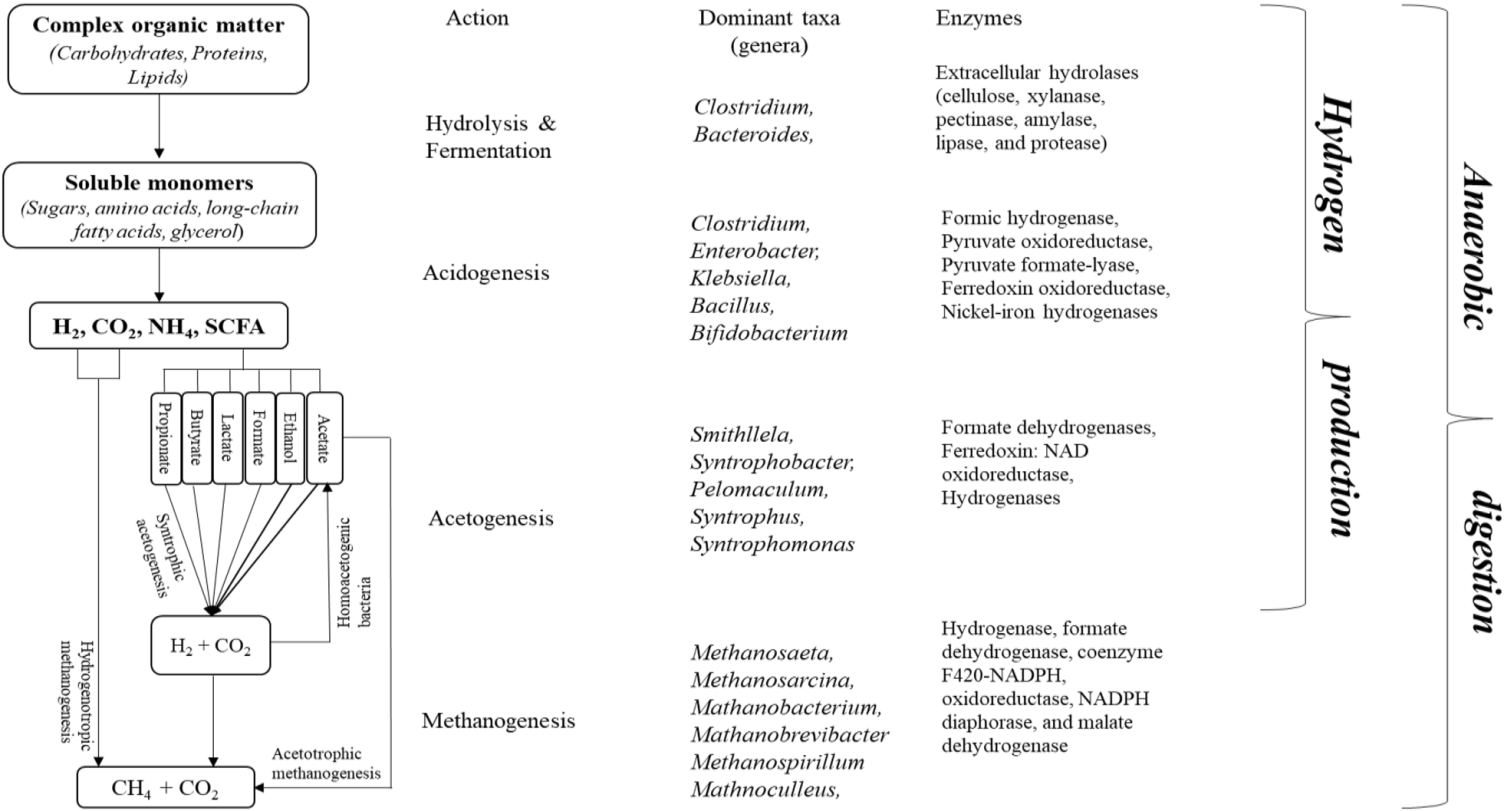
Key enzymes and dominant microbial taxa involved during anaerobic digestion of organic matter

The main biochemical pathways in dark fermentation overlaps with those of anaerobic digestion, where diverse microbial communities synergistically work together to ensure a stable degradation of organic substrates (Abendroth et al. 2015; Stolze et al. 2016). The pathways can be divided into four phases: hydrolysis, acidogenesis, acetogenesis, and methanogenesis (Fig. 1). In anaerobic digestion, hydrogen (H_2_) is produced during acidogenesis and acetogenesis, by hydrolytic and fermentative bacteria. It is later consumed during methanogenesis, when methanogenic archaea use H_2_ and CO_2_ to produce methane (CH_4_) (Hassa et al. 2018). Therefore, inhibition of methanogenesis is necessary to re-direct the pathway for hydrogen production, through the final step of dark fermentation. The initial hydrolysis starts when the complex substrates (polysaccharides, lipids, and proteins) are hydrolysed to monomers (sugars, amino acids, fatty acids) by the actions of extracellular hydrolytic enzymes such as cellulase, pectinase, lipase and protease. The microbial taxa responsible for the hydrolytic activities are mainly represented by two phyla, *Firmicutes* and *Bacteroidetes*, from the genera *Clostridium* and *Bacteroides*. The abundance of these phyla can be attributed to their degradative abilities, as the main degraders of cellulolytic materials (Abendroth et al. 2015). Members of these phyla are also known to be fast growers, utilising the hydrolysed products for growth through fermentation, and are usually present in the whole degradation process. They are also less sensitive to changes in environmental conditions (Li et al. 2017; Wang et al. 2018). The hydrolysis step is usually not a rate-limiting step, except with recalcitrant substrates such as lignocellulosic waste, which usually results in incomplete hydrolysis requiring a pre-treatment step (Menzel et al. 2020).

Next, in acidogenesis, the hydrolysed products are further metabolised to H_2_, CO_2_, and short-chain fatty acids (SCFA) (e.g. acetate, butyrate, formate, propionate, etc.) by acidogenic microbial communities. The predominant phyla associated with this phase are *Bacteroidetes*, *Firmicutes*, *Chloroflexi*, and *Proteobacteria* (Audu et al. 2021; Castellano-Hinojosa et al. 2018). Acidogenesis is usually a rapid process, accompanied by the accumulation of SCFA and the subsequent drop in pH. The microorganisms in the acidogenic phase consist of both facultative and obligate anaerobes and are often referred to as acidogens, or acid formers. The commonly reported genera participating in this phase are *Clostridium*, *Bacteroides*, *Bifidobacterium*, *Bacillus*, and *Streptococcus* (Gonzalez-Martinez et al. 2016; Seon et al. 2014). In this phase, carbohydrates (mostly glucose, the preferred substrates) are converted to pyruvate through the glycolytic pathway (Saravanan et al. 2021; Vardar‐Schara et al. 2008). Under mesophilic condition, the H_2_-yielding fermentation routes are the obligate anaerobic (*Clostridium* type) and facultative anaerobic (*Enterobacteria* type) fermentation route.

**Fig. 2:**
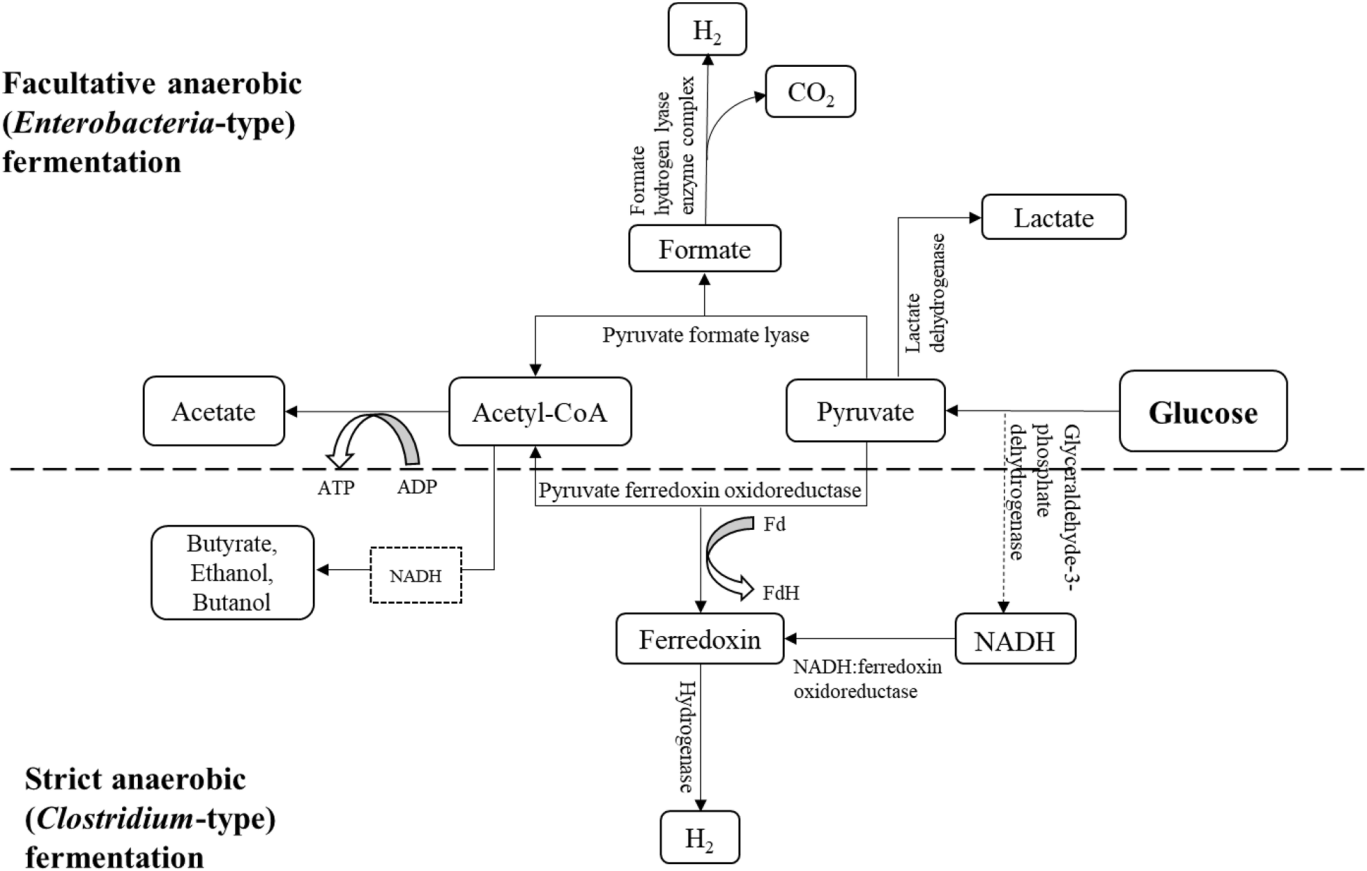
An overview of the metabolic pathways of acidogenesis

In the facultative anaerobic fermentation route, pyruvate is further converted to acetyl-CoA and formate, by pyruvate formate lyase (PFL), and H_2_ is produced from formate by the formate hydrogen lyases enzyme complex. The strict anaerobic fermentation route involves the oxidisation of pyruvate to acetyl-CoA and reduced ferredoxin (Fd) by pyruvate ferredoxin oxidoreductase (PFOR). H_2_ is then released from the reduced Fd by the action of hydrogenase. Additional molecules of H_2_ can also be produced from the oxidisation of nicotinamide adenine dinucleotide (NADH) to reduced Fd by NADH:ferredoxin oxidoreductase (NFOR), followed by the subsequent release of H_2_ from the reduced Fd by hydrogenase (Fig. 2). However, the activities of NFOR is usually inhibited under standard conditions and can only proceed when H_2_ partial pressure is very low, as opposed to PFOR which is still active at standard H_2_ partial pressure (Kraemer and Bagley 2007). In addition to H_2_, acetyl-CoA can also be further converted to non-gaseous products including SCFA (acetate, lactate, butyrate, propionate), alcohols (ethanol, butanol), and ketones (acetone). The overall theoretical maximum yield of H_2_ from the reduced Fd pathway is 4 mol of H_2_ per 1 mol of glucose, and 2 mol of H_2_ per 1 mol of glucose from the formate pathway. The yield is influenced by the fermentation end products generated alongside H_2_. Theoretically, based on the ‘Thauer limit’, the maximum yield of 4 mol of H_2_ can be obtained with acetate as the fermentation end product, 2 mol with butyrate or propionate, and much lower yields when alcohols are the end products (Keskin et al. 2019; Vardar‐Schara et al. 2008).

The intermediates products H_2_, CO_2_, and acetate can directly be utilised by methanogens for methane production, while other products such as butyrate, propionate, valerate require further transformation or conversion through syntrophic acetogenesis first (Lim et al. 2020). In the acetogenesis phase, unusable substrates are converted to acetate, CO_2_, and H_2_ by hydrolytic and fermentative bacteria that do not possess hydrolytic activities. Acetogenesis is also the rate-limiting phase. In addition, the H_2_ produced from acetogenesis is converted to CH_4_ by the hydrogenotrophic methanogens (Venkiteshwaran et al. 2015). The oxidation of non-gaseous products of acidogenesis is based on the reverse electron transfer process, a thermodynamically unfavourable condition. The process requires energy input to drive the oxidation/reduction process involving multiple enzyme systems, such as formate dehydrogenases, ferredoxin:NAD oxidoreductases, hydrogenases, reactive quinone complexes, *c*-type cytochromes, etc. (Sieber et al. 2012). However, when oxidation is coupled with methane production, energy conversion is more feasible due to the diminishing effects of H_2_ pressure created by the methanogenic activity (Sikora et al. 2017). The most commonly reported syntrophic acetogens in anaerobic digester are the propionate degraders belonging to the genera *Pelotomaculum*, *Smithllela*, and *Syntrophobacter*. While the oxidation of butyrate and other fatty acids is carried out by *Syntrophus* and *Syntrophomonas* (Venkiteshwaran et al. 2015). The acetogenesis phase is important because it ensures rapid and stable anaerobic digester operation by preventing methanogenic inhibition due to the high acid concentrations (Wang et al. 2018).

Methanogenesis is the final phase in anaerobic digestion, in which acetate, H_2_ and CO_2_ produced from the acidogenic and acetogenic phases are further transformed into biogas, in the form of CH_4_ and CO_2_. Methanogens are the main hydrogen consumers in the anaerobic environments, and for this reason they are usually suppressed in biohydrogen dark fermentation to maximise the hydrogen yield. Unlike the previous three phases which are dominated by fermentative bacteria, the methanogenesis phase is exclusively dominated by the methanogenic archaea. The methanogens are slow growers and sensitive to environmental changes. Methanogenesis can occur via three possible pathways based on the available substrate: acetoclastic, methylotrophic, or hydrogenotrophic methanogenesis. Acetotrophic/acetoclastic methanogens use acetate as substrate by catalysing its methane production, methylotrophic methanogens use methyl-based compounds, and hydrogenotrophic methanogens use CO_2_ and H_2_ for CH_4_ production (Hassa et al. 2018; Lim et al. 2020; Menzel et al. 2020). The commonly observed methanogens associated with biogas production are from the genera *Methanosaeta* and *Methanosarcina* (acetotrophic methanogens); and *Methanobacterium*, *Methanospirillum*, *Methanococcus*, and *Methanobrevibacter* (hydrogenotrophic methanogens) (Castellano-Hinojosa et al. 2018). The acetotrophic methanogens have been reported to be the most predominant type of methanogen in anaerobic digesters, and are responsible for about 70% of the methane generated (Gonzalez-Martinez et al. 2016). The genus *Methanosaeta* for example, are obligate acetoclastic methanogens, characterised as slow growers, and only use acetate as the substrate. While *Methanosarcina* are facultative acetoclastic methanogens, have faster growth rate, and can utilise a wider substrate range in addition to acetate. Unlike *Methanosaeta*, members of the *Methanosarcina* genus have a low affinity to acetate, which can account for its abundance in high acetate concentration condition by outgrowing the *Methanosaeta* population. At low acetate concentration, *Methanosaeta* have been reported to dominate the archaea community. Due to their high affinity to acetate, *Methanosaeta* genus outcompete *Methanosarcina* population by utilising the available acetate in the environment (Conklin et al. 2006; Lim et al. 2020).

The intricacy and complexity of dark fermentation makes it a process “black box”, due to the variety of multiple metabolic activities and interactions within the microbial community, along with the limited biogas-producing microbial genomes in the reference databases. So far, biohydrogen yield obtained in practice are mostly up to 32%, hampered by the Thauer limit (Patel et al. 2018). In addition, biohydrogen production from dark fermentation of organic wastes seldom exceeded 2 mol H_2_/mol hexose (Wang and Yin 2019). A number of approaches have been explored to overcome the bottleneck of dark fermentative biohydrogen production, including reactor configurations, operational condition, inoculum and substrate types, pre-treatment strategies and integrating multiple biohydrogen production systems (Audu et al. 2020). The microbiomes of the systems are the integral part of these processes. Identifying the microorganisms and understanding the behaviour is crucial to dark fermentation robustness, as elaborated in sections "Industrial wastes as substrates" and "Palm oil mill effluent (POME) as substrate".

### Industrial wastes as substrates

Dark fermentative biohydrogen production has been investigated using a diverse range of pure cultures (Table 1). *Clostridium butyricum* represents the most commonly studied pure culture under mesophilic condition. *C. butyricum* is known for its high biohydrogen yield regardless of the substrates complexity, which can range from simple carbohydrates such as glucose, xylose and sucrose, to complex biomass such as food waste (Kanchanasuta et al. 2017), glycerol (Kivistö et al. 2013; Yin and Wang 2017), microalgae (Ortigueira et al. 2015) and sugarcane bagasse (Plangklang et al. 2012). At present, the highest reported biohydrogen yield from the conversion of organic waste by *C. butyricum* was 3.0 mol H_2_/mol glycerol which is equal to 3.6 mol H_2_/mol hexose out of the theoretical stoichiometric yield (4 mol H_2_/mol hexose, the Thauer limit) when fermenting raw glycerol from biodiesel production process (Kivistö et al. 2013). In addition, other *Clostridia* species including *C. beijerinckii* (Rambabu et al. 2021), *C. pasteurianum* (Sarma et al. 2019), *C. acetobutylicum* (Azman et al. 2016) and *C. saccharoperbutylacetonicum* (Dada et al. 2013) have also been investigated for biohydrogen production from organic wastes under mesophilic condition. Under thermophilic conditions, *C. thermocellum* has been reported to be the ideal strain. Rambabu et al. (2020) obtained 103.97 mmol H_2_/L using *C. thermocellum* to produce biohydrogen from date seeds waste through dark fermentation system operated at 50 °C and initial pH 7. Tian et al. (2015) also used *C. thermocellum* to ferment sugarcane bagasse at 55 °C and obtained 4.89 mmol H_2_/g medium added. Versatile substrate utilisation with high biohydrogen yields of 0.23–3.47 H_2_/mol hexose from *C. butyricum* and 0.52–3.0 mol H_2_/mol hexose from the other *Clostridium* species have made *Clostridia* popular for use in dark fermentation (Wang and Yin 2019). However, the strict anaerobic requirement of *Clostridia* complicates their practical applications.

**Table 1:** Dark fermentative biohydrogen production from various substrates (including industrial wastes) using pure culture

Microorganism	Substrate	Reactor type	Operating conditions		Biohydrogen yield	References
			Temperature, *T*	pH		
*Clostridium butyricum* INET 1	Glucose	Batch	35 °C	Initial = 7.0, Operation = uncontrolled	2.24 mol H_2_/mol hexose	Yin and Wang (2017)
*Clostridium butyricum* INET 1	Xylose	Batch	35 °C	Initial = 7.0, Operation = uncontrolled	1.23 mol H_2_/mol hexose	Yin and Wang (2017)
*Clostridium butyricum* INET 1	Sucrose	Batch	35 °C	Initial = 7.0, Operation = uncontrolled	1.44 mol H_2_/mol hexose	Yin and Wang (2017)
*Clostridium butyricum* INET 1	Lactose	Batch	35 °C	Initial = 7.0, Operation = uncontrolled	1.83 mol H_2_/mol hexose	Yin and Wang (2017)
*Clostridium butyricum* INET 1	Starch	Batch	35 °C	Initial = 7.0, Operation = uncontrolled	2.17 mol H_2_/mol hexose	Yin and Wang (2017)
*Clostridium butyricum* INET 1	Glycerol	Batch	35 °C	Initial = 7.0, Operation = uncontrolled	0.67 mol H_2_/mol hexose	Yin and Wang (2017)
*Clostridium butyricum* TISTR 1032	Food waste	CSTR	37 °C	Initial = 6.0, Operation = uncontrolled	362 mL H_2_/g VS	Kanchanasuta et al. (2017)
*Clostridium butyricum* DSM 10,702	Microalgae	Batch	37 °C	ND	2.74 mol H_2_/mol glucose	Ortigueira et al. (2015)
*Clostridium butyricum* CWBI 1009	Glucose	TBSBR	30 °C	Initial = 5.2, Operation = 5.2	1.67 mol H_2_/mol glucose	Puhulwella et al. (2014)
*Clostridium butyricum*	Glycerol	Batch	37 °C	Initial = 7.4, Operation = uncontrolled	3.0 mol H_2_/mol glycerol	Kivistö et al. (2013)
*Clostridium butyricum* CWBI 1009	Glucose	AnSBR	30 °C	Initial = 7.6, Operation = uncontrolled	2.2 mol H_2_/mol glucose	Beckers et al. (2013)
*Clostridium butyricum* CWBI 1009	Glucose	ABR	30 °C	Initial = 8.5, Operation = uncontrolled	2.49 mol H_2_/mol glucose	Laurent et al. (2012)
*Clostridium butyricum* TISTR 1032	Sugarcane bagasse	Serum bottle	37 °C	Initial = 6.5, Operation = 6.5	1.52 mol H_2_/mol hexose_used_	Plangklang et al. (2012)
*Clostridium beijerinckii* DSM 791	Rice mill wastewater	Serum bottle	37 °C	Initial = 7.0, Operation = uncontrolled	214.9 mL H_2_/L	Rambabu et al. (2021)
*Clostridium beijerinckii* PS-3	Oil palm sap	Serum bottle	30 °C	Initial = 7.0, Operation = uncontrolled	141 mL H_2_/g substrate	Noparat et al. (2012)
*Clostridium pasteurianum* DSM 525	Glycerol	Serum bottle	37 °C	Initial = 7.0, Operation = 7.0	1.10 mol H_2_/mol glycerol	Sarma et al. (2019)
*Clostridium pasteurianum*	Glucose	Serum bottle	35 °C	Initial = 7.0, Operation = uncontrolled	2.2 mol H_2_/mol xylose	Hsieh et al. (2016)
*Clostridium* BOH3	Fruit waste	Serum bottle	37 °C	Initial = 6.8, Operation = uncontrolled	359.97 mL H_2_/g TS utilised	Mahato et al. (2020)
*Clostridium thermocellum* ATCC 27,405	Date seeds	Serum bottle	50 °C	Initial = 7.0, Operation = uncontrolled	103.97 mmol H_2_/ L	Rambabu et al. (2020)
*Clostridium acetobutylicum* YM1	Rice bran	Batch	34 °C	Initial = 5.5, Operation = uncontrolled	117.24 mL H_2_/g sugar_consumed_	Azman et al. (2016)
*Clostridium thermocellum* ATCC 27,405	Sugarcane bagasse	Serum bottle	55 °C	Initial = 6.6, Operation = uncontrolled	4.89 mmol H_2_/g medium_added_	Tian et al. (2015)
*Clostridium saccharoperbutylacetonicum* N1-4	Rice bran	Batch	30 °C	Initial = 6.0, Operation = uncontrolled	3.37 mol H_2_/mol sugar_consumed_	Dada et al. (2013)
*Clostridium tyrobutyricum* Fya102	Glucose	CSTR	35 °C	Initial = 6.0, Operation = 6.0	1.06 mmol H_2_/mmol glucose	Whang et al. (2011)
*Enterobacter aerogenes* ZJU1	Aquatic weed	Batch	37 °C	Initial = 6.0, Operation = uncontrolled	62.2 mL H_2_/g VS	Song et al. (2020)
*Enterobacter asburiae*	Lactose	Batch	25.6 °C	Initial = 7.2, Operation = uncontrolled	1.19 mol H_2_/mol lactose	Alvarez-Guzmán et al. (2020)
*Enterobacter aerogenes* CDC 819–56	Sago wastewater	Serum bottle	31 °C	Initial = 6.8, Operation = uncontrolled	7.42 mmol H_2_/mol glucose	Ulhiza et al. (2018)
*Enterobacter aerogenes* PTCC 1221	Rice straw	Serum bottle	37 °C	Initial = 5.8, Operation = 5.8	19.73 mL H_2_/g straw	Asadi and Zilouei (2017)
*Enterobacter cloacae* IIT-BT 08	Distillery effluent	Serum bottle	37 °C	Initial = 7.5, Operation = uncontrolled	7.38 mol H_2_/kg COD_reduced_	Mishra and Das (2014)
*Bacillus cereus*	Wheat straw	Batch	37 °C	Initial = 7.5, Operation = uncontrolled	156.4 mL H_2_/g VS	Saleem et al. (2020)
*Ethanoligenens harbinense* B49	Glucose	Serum bottle	36 °C	Initial = 6.5, Operation = uncontrolled	113.5 mmol H_2_/L	Xu et al. (2016)
*Ethanoligenens harbinense* YUAN-3	Glucose	Batch	35 °C	Initial = 7.0, Operation = 4.5	2.62 mol H_2_/mol glucose	Zhang et al. (2015)
*Escherichia coli*	Glucose	Serum bottle	37 °C	ND	2.0 mol H_2_/mol glucose	Bisaillon et al. (2006)
*Janthinobacterium agaricidamnosum*	Glucose	Serological bottle	25 °C	Initial = 6.5, Operation = uncontrolled	0.86 mol H_2_/mol glucose	Alvarado-Cuevas et al. (2015)
*Polaromonas jejuensis*	Glucose	Serological bottle	25 °C	Initial = 6.5, Operation = uncontrolled	1.57 mol H_2_/mol glucose	Alvarado-Cuevas et al. (2015)
*Klebsiella pneumoniae*	Brewery wastewater	AnBBR	35–36 °C	Initial = 5.5, Operation = uncontrolled	0.80–1.67 mol H_2_/mol glucose	Estevam et al. (2018)
*Klebsiella pneumoniae* BLb01	Glycerol	Batch	39 °C	Initial = 9.0, Operation = uncontrolled	45.0 mol %	Costa et al. (2011)

ABR: anaerobic biodisc reactor; AnBBR: mechanically stirred anaerobic reactor; AnSBR: anaerobic sequenced-batch reactor; CSTR: continuous stirred tank reactor; TBSBR: trickling-bed sequenced-batch reactor; ND: no data; COD: chemical oxygen demand; TS: total solid; VS: volatile solid

Facultative anaerobes such as *Enterobacter*, *Klebsiella*, *Citrobacter*, *Escherichia* and *Bacillus* are the alternative candidates for biohydrogen dark fermentation. These species possess the ability to shift from aerobic respiration producing adenosine triphosphate (ATP) in the presence of oxygen, to fermentation in the absence of oxygen (Łukajtis et al. 2018). Nevertheless, facultative anaerobes generally produce lower biohydrogen yield than the strict anaerobes, e.g. *Clostridia*, but the high tolerance to oxygen makes them more attractive for practical applications. Pure cultures have commonly been used for investigations on biohydrogen production and the related metabolic activity. This allows the investigation into the metabolic pathways involved, and subsequently the feasible ways to enhance the biohydrogen production efficiency through metabolic engineering (Wang and Yin 2019). In addition, reliable biohydrogen yields by avoiding the production of undesired by-products, reproducible bioprocess and ease of genetic manipulations are the other benefits of using pure cultures (Kumar et al. 2018). However, aseptic condition is compulsory to handle pure cultures as they are highly susceptible to contaminations. They are also substrate selective, and developing pure cultures to reach the optimal production period can be time consuming (Kumar et al. 2018).

Mixed cultures have also been widely used (Table 2). Inoculum pre-treatment is necessary in a mixed culture system to enhance biohydrogen production yield by suppressing the activity of competing species, such as the biohydrogen-consumers and substrates competitor. Different pre-treatment methods will result in different starting microbial community structures. Zhang et al. (2011) investigated the effects of five different inoculum pre-treatment methods on mixed culture of aerobic seed sludge to enhance biohydrogen production from corn stover hydrolysate. Inoculum with no pre-treatment is composed of complex microbial community mainly represented by *Enterobacter* sp. and *Pantoea* sp. Pre-treatment using heat achieved the highest biohydrogen yield, with the microbial community in the fermentation system dominated by *C. bifermentans*. Pre-treatment using base, acid, ultrasonic disruption and ultraviolet radiation favours facultative anaerobes, such as *E. aerogenes*, *Klebsiella*, *Pectobacterium* and *E*. *coli*. Heat pre-treatment is the commonly used inoculum pre-treatment method under mesophilic conditions (Table 2). It is usually selective to spore-forming species such as *Clostridia,* and inhibits other non-spore formers. In general, the use of mixed culture in dark fermentation has been shown to be promising, and it offer high hydrogen evolution rate and yields (Pachapur et al. 2019). However, understanding the metabolic complexities and process kinetics taking place within undefined microbiome systems are challenging.

**Table 2:** Dark fermentative biohydrogen production from industrial wastes using mixed culture

Inoculum source	Substrate	Inoculum pre-treatment	Reactor type	Operating Conditions		% H_2_	H_2_ yield	H_2_ production rate	Dominant members	Technique for microbial community analysis	References
				Temperature, *T*	pH						
Wastewater treatment plant	Glycerol	IR	Batch	36 °C	Initial = 7.0, Operation = uncontrolled	ND	0.52 mol H_2_/mol glycerol	ND	ND	ND	Chen et al. (2021)
Sewage treatment plant	Glucose	IR	Batch	36 °C	Initial = 7.0, Operation = uncontrolled	ND	ND	ND	*Clostridium *sensu stricto 1 and *Clostridium butyricum*	Amplicon sequencing—Illumina MiSeq-PICRUSt	Yin and Wang (2021)
Biogas slurry	Sake lees	HT, 150 °C, 40 min	Batch	37 °C	Initial = 6.0, Operation = uncontrolled	ND	112.07 mL H_2_/g COD	ND	*Pantosa agglomerans*, *Clostridium acetobutylicum*, *Clostridium butyricum*	Amplicon sequencing—Illumina MiSeq	Choiron et al. (2020)
Biogas digester of pig farm	Alligator weed	HT, 100 °C, 30 min	Batch	37 °C	Initial = 7.0, Operation = uncontrolled	ND	48.4 mL H_2_/g VS	5.63 mL H_2_/g VS h	*Clostridium *sensu stricto 1, *Anaerobacterium*, *Clostridium IV*	Amplicon sequencing—Illumina MiSeq-gene function prediction	Li et al. (2020)
Chicken manure	Cassava	HT, 95 °C, 15 min	Hungate tube	36 °C	Initial = 6.0, Operation = uncontrolled	ND	ND	16.62 mL H_2_/L h	Clostridiaceae, Porphyromonadaceae and Rimonococcaceae	Amplicon sequencing—Illumina MiSeq	Martinez-Burgos et al. (2020)
Vinasse effluent	Cassava	HT, 95 °C, 15 min	Hungate tube	37 °C	Initial = 6.0, Operation = uncontrolled	ND	ND	21.82 mL H_2_/L h	Clostridiaceae, Porphyromonadaceae and Rimonococcaceae	Amplicon sequencing—Illumina MiSeq	Martinez-Burgos et al. (2020)
Indigenous microbes	Banana wastes	ND	Batch	37 °C	Initial = 7.5, Operation = uncontrolled	ND	38.08 mL H_2_	ND	*Lactobacillus* and *Clostridium*	Metagenomic—Illumina HiSeq	Mazareli et al. (2020)
Indigenous microbes	Coffee wastes	ND	Batch	30 °C	Initial = 7.0, Operation = uncontrolled	ND	240 mL H_2_	3040 mL H_2_/L day	*Clostridium* sp., *Lactobacillus* sp., *Kazachstania* sp. and *Saccharomyces* sp.	Metagenomic—Illumina NovaSeq	Villa Montoya et al. (2020)
Indigenous microbes	Sugarcane molasses	ND	AnSTBR-A	55 °C	Initial = 3.8, Operation = uncontrolled	51	1.18 mol H_2_/mol total carbohydrates	88 mL H_2_/L h	*Thermoanaerobacterium*	Amplicon sequencing—Ion Torrent	Oliveira et al. (2020)
Effluent of hydrogen fermenter	Vegetable waste	ND	Batch	35 °C	Initial = 7.0, Operation = uncontrolled	43.54	151.67 mL H_2_/g VS_added_	ND	ND	ND	Panin et al. (2020)
Soil sediment of mangroves	Cassava pulp	ND	Serum bottle	60 °C	Initial = 7.0, Operation = uncontrolled	ND	23 mL H_2_/g substrate	ND	*Clostridium thermopalmarium*, *Clostridium isatidis, Thermoanaerobacterium*, *Fonticella tunisiensis*	Amplicon sequencing—Illumina	Pason et al. (2020)
Municipal wastewater treatment plant	Glycerol	ND	CSTR	37 °C	Initial = 6.0, Operation = 6.5	49	403.6 mmol H_2_/mol Gly_consumed_	49.4 mL H_2_/L h	*Clostridium intestinale*	Amplicon sequencing—Illumina MiSeq	Paillet et al. (2019)
Sewage treatment plant	Glucose	HT, 100 °C, 15 min	Batch	37 °C	Initial = 7.0, Operation = uncontrolled	ND	1.4 mol H_2_/mol glucose	ND	*Clostridium *sensu stricto 1 (*C. butyricum* and *C. paraputrificum*), *Paraclostridium*, *Paeniclostridium* and *Romboutsia*	Amplicon sequencing—Illumina MiSeq	Yang and Wang (2019)
Buffalo sludge and rumen	Buffalo waste	ND	Batch	39 °C	Initial = 7.0, Operation = uncontrolled	48.1	120.8 mL H_2_/g VS	ND	*Clostridia Incertae Sedis*, *Clostridium *sensu stricto 1, *Prevotella*	Amplicon sequencing—Illumina MiSeq	Chiariotti and Crisà (2018)
Food waste treatment plant	Glucose	No pre-treatment	UASB	38 °C	Initial = 4.9, Operation = uncontrolled	43.49	0.89 mol H_2_/mol hexose	4.68 L H_2_/L day	*Enterobacter cloacae*, *Aeromonas hydrophila*, *Clostridium pasteurianum*	Amplicon sequencing—Ion Torrent	Cho et al. (2018)
Mixed culture	Tequila vinasse	ND	CSTR	35 °C	Initial = 6.5, Operation = 5.8	70	124 NmL H_2_/g VS_added_	159 NmL H_2_/L h	*Clostridium beijerinckii*, *Streptococcus* sp. and *Acetobacter lovaniensis*	Amplicon sequencing—Illumina MiSeq	García-Depraect and León-Becerril (2018)
Soil and composting residue	Sugarcane bargasse	ND	Batch	37 °C	Initial = 6.0, Operation = uncontrolled	ND	1.53 mmol H_2_/L	ND	*Clostridium, Bacteroides*, *Parabacteroides*, *Porphyromonas*, *Desulfitobacterium*, *Bacillus* and *Methanothermobacter*	Metagenomic—Illumina HiSeq	Soares et al. (2018)
Anaerobic sludge	Food waste	ND	Batch	35 °C	Initial = 6.3, Operation = 7.0					Metaproteomic	Jia et al. (2017)
Brewery wastewater treatment plant	Glucose	HT, 90 °C, 30 min	FBR	37 °C	Initial = 5.5, Operation = 5.5	ND	2.3 mol H_2_/mol glucose_added_	78 L H_2_/L day	*Clostridium butyricum*, *Enterococcus* sp. and *Enterobacter* sp.	qPCR	Pugazhendhi et al. (2017)
Domestic wastewater treatment plant	Glycerol	Ae, 24 h	CSTR	37 °C	Initial = 5.5, Operation = 5.5	61	0.58 mol H_2_/mol glycerol	88.0 mmol H_2_/L day	*Clostridium* sp., *Prevotella* sp. and *Klebsiella* sp.	CE-SSCP—Illumina MiSeq	Silva-Illanes et al. (2017)
Domestic wastewater treatment plant	Glycerol	Ae, 24 h	CSTR	37 °C	Initial = 6.0, Operation = 6.0	57	0.26 mol H_2_/mol glycerol	51.0 mmol H_2_/L day	*Clostridium* sp., *Enterococcus* sp., *Prevotella* sp. and *Snodgrassella* sp.	CE-SSCP—Illumina MiSeq	Silva-Illanes et al. (2017)
Brewery industry	Food waste	HT, 103–105 °C, 60 min	ASBR	35 °C	Initial = 5.5, Operation = 5.5	37.9	103.6 mL H_2_/g COD_removed_	226.4 mL H_2_/L h	*Megasphera*, *Veillonella*	Pyrosequencing—Roche	Moreno-Andrade et al. (2015)
Fruit juice wastewater treatment plant	Food waste	HT, 100 °C, 60 min	Batch	37 °C	Initial = 6.0, Operation = uncontrolled	ND	2.68 mol H_2_/mol hexose	ND	*Clostridium frigidicarnis*	Pyrosequencing—Roche	Laothanachareon et al. (2014)
Sugarcane stillage treatment plant	Sugarcane stillage	HT, 90 °C, 10 min	AFBR	55 °C	ND	43.3–48.9	2.23 mmol H_2_/g COD_added_	1.49 L H_2_/L h	*Megasphera* sp., *Lactobacillus*	PCR-DGGE—Cloning	Santos et al. (2014)
Garbage compost	Beer lees	No pre-treatment	Batch	37 °C	Initial = 4.1–7.0, Operation = uncontrolled	ND	29.3 mL H_2_/g TS	ND	*Clostridium roseum*	PCR-DGGE	Bando et al. (2013)
Municipal wastewater treatment plant	Rice straw hydrolysate	HT, 95–100 °C, 60 min	CSABR	37 °C	Initial = 5.5, Operation = 5.5	ND	0.69 mol H_2_/mol T-sugar	10 L H_2_/L day	*Clostridium pasteurianum*	PCR-DGGE	Liu et al. (2013)
Beach	Sucrose	HT, 100 °C, 45 min	AGSBR	35 °C	Initial = 6.0, Operation = 6.0	37	1.04 mol H_2_/mol sucrose	15.59 m^3^ H_2_/ m^3^ day	*Clostridium pasteurianum* and *Bifidobacteria* sp.	PCR-DGGE	Lin et al. (2011)
Wastewater treatment plant	Corn stover hydrolysate	HT, 100 °C, 60 min	Serum bottle	37 °C	Initial = 7.0, Operation = uncontrolled	ND	502 mL H_2_/L	37.3 mL H_2_/h	*Clostridium bifermentans*, *Escherichia coli*, *Escherichia vulneris*, Uncultured *Escherichia* sp.	PCR-DGGE—Cloning	Zhang et al. (2011)
Wastewater treatment plant	Corn stover hydrolysate	Ac, pH 3, 60 min	Serum bottle	37 °C	Initial = 7.0, Operation = uncontrolled	ND	352.1 mL H_2_/L	13.7 mL H_2_/h	*Enterobacter aerogenes*, *Escherichia coli*, *Escherichia vulneris*, Uncultured *Escherichia* sp.	PCR-DGGE—Cloning	Zhang et al. (2011)
Wastewater treatment plant	Corn stover hydrolysate	Ba, pH 12, 60 min	Serum bottle	37 °C	Initial = 7.0, Operation = uncontrolled	ND	458.4 mL H_2_/L	12.3 mL H_2_/h	*Enterobacter aerogenes*, *Klebsiella*, *Pectobacterium* sp.	PCR-DGGE—Cloning	Zhang et al. (2011)
Wastewater treatment plant	Corn stover hydrolysate	Us, 15 min	Serum bottle	37 °C	Initial = 7.0, Operation = uncontrolled	ND	295.9 mL H_2_/L	18.7 mL H_2_/h	*Enterobacter aerogenes*, *Pectobacterium* sp.	PCR-DGGE—Cloning	Zhang et al. (2011)
Wastewater treatment plant	Corn stover hydrolysate	Uv, 15 min	Serum bottle	37 °C	Initial = 7.0, Operation = uncontrolled	ND	290.9 mL H_2_/L	58.9 mL H_2_/h	*Pectobacterium* sp.	PCR-DGGE—Cloning	Zhang et al. (2011)
Sewage treatment plant	Molasses	HT, 100 °C, 45 min	CSTR	35 °C	Initial = 5.5, Operation = 5.5	47	2.1 mol H_2_/mol hexose	153 mmol H_2_/L day	*Clostridium acetobutylicum* and *Clostridium pasteurianum*	qPCR	Lay et al. (2010)

HT: heat treatment; IR: ionising radiation; Ac: acid; Ae: aeration; Ba: base; ND: no data; Us: ultrasonication; Uv: ultraviolet; AFBR: anaerobic thermophilic fluidised bed reactor; AGSBR: agitated granular sludge bed reactor; AnSTBR-A: anaerobic structured-bed reactor; ASBR: anaerobic sequencing batch reactor; CSABR: continuously stirred anaerobic bioreactor; CSTR: continuous stirred tank reactor; FBR: fixed-bed reactor; UASB: up-flow anaerobic sludge blanket reactor; COD: chemical oxygen demand; Gly: glycerol; VS: volatile solid; CE-SSP: capillary electrophoresis single-stranded conformation; PCR-DGGE: polymerase chain reaction-denaturing gradient gel electrophoresis; qPCR: quantitative polymerase chain reaction

Artificial microbial consortia containing selected microorganisms with specific metabolic or ecological functions has been shown to overcome the limitations of wild type and undefined microbiomes (Ergal et al. 2020). Recently, precision design of an artificial microbial consortia consisting *E. aerogenes* and *C. acetobutylicum* yielded 5.6 mol H_2_/mol glucose. This was the highest biohydrogen yield reported so far, 40% beyond the Thauer limit (Ergal et al. 2020). The finding suggests that constructing a desired microbial consortium with well-studied biohydrogen-producing species will enable a comprehensive understanding of the microbial interactions, ease the control and balancing the effects of any perturbations. This will ultimately create a more efficient and robust engineered system.

### Palm oil mill effluent (POME) as substrate

POME is the wastewater produced in large quantity during palm oil processing. It contains substantial amount of organic material, suspended solids, and oil and greases. Despite its nontoxic nature, POME is categorised as extremely high strength wastewater, which is 100 times more polluted than municipal sewage, and require effective treatment before discharge into the environment (Chia et al. 2020). Raw POME appears as thick brownish high colloidal suspension liquid mixture with a distinct offensive odour (Chia et al. 2020). It is characterised by high biological oxygen demand (BOD) (10, 250–80, 400 mg/L), high chemical oxygen demand (COD) (15,000–100,000 mg/L), high oil and grease content (130–18,000 mg/L), high suspended solids (5000–54,000 mg/L), high discharge temperature (50–90 °C) and is acidic (pH 3.4–6.9) (Audu et al. 2020). POME is rich with organic materials containing cellulose (11%), hemicellulose (7%) and lignin (42%) (O-Thong et al. 2012). Given the high organic matter properties, recent POME treatment methods are coupled with bioenergy production and other value-added products, such as solvents, biomethane and biohydrogen.

*Clostridia* is the most commonly used genera for biohydrogen production from POME. *C. butyricum* has been used in several studies as pure culture inoculum for mesophilic batch biohydrogen production from POME via dark fermentation (Table 3). Singh et al. (2013b) observed that biohydrogen yield increased 1.5- to 2-fold when using an acclimatised immobilised *C. butyricum*. The immobilised cells recorded a biohydrogen yield of 5350 mL H_2_/L POME with maximum biohydrogen production rate of 510 mL H_2_/L POME/h. This species has also been reported to be the dominant biohydrogen producer in POME fermentation using mixed culture (Yossan et al. 2012) (Table 4). The effects of mesophilic and thermophilic conditions were also investigated, using anaerobic sludge as inoculum. Higher biohydrogen yield was achieved from mesophilic fermentation (27.09 mL/g COD) while higher biohydrogen production rate was achieved under thermophilic condition (49.34 mL H_2_/L POME/h). Microbial community analysis performed showed that *Clostridia* dominated all the biohydrogen production systems operated at 25, 37, 45 and 55 °C.

**Table 3:** Dark fermentative biohydrogen production from POME using pure culture

Inoculum	Reactor type	Operating conditions		Biohydrogen yield	References
		Temperature, *T*	pH		
*Clostridium beijerinckii*	Hungate tube	30 °C	Initial = 7.0, Operation = uncontrolled	4620 mL H_2_/L medium	Rosa et al. (2020)
*Bacillus anthracis* PUNAJAN 1	CSTR	35 °C	Initial = 6.5, Operation = uncontrolled	236 ml H_2_/g COD	Mishra et al. (2017)
*Escherichia coli*	Serum bottle	37 °C	Initial = 8.5, Operation = uncontrolled	0.66 mol H_2_/mol total monomeric sugars	Taifor et al. (2017)
*Clostridium* LS2	UASB	37 °C	Initial = 5.5, Operation = 5.5	380 mL H_2_/g COD	Singh et al. (2013b)
*Clostridium butyricum* EB6	Batch	37 °C	Initial = 5.5, Operation = 5.5	5350 mL H_2_/L POME	Singh et al. (2013c)
*Clostridium butyricum*	Batch	37 °C	Initial = 7.0, Operation = 5.5	2.18 mol H_2_/mol total carbohydrate	Kamal et al. (2011)
*Clostridium butyricum* EB6	Batch	37 °C	Initial = 5.5, Operation = 5.5	3195 mL H_2_/L POME	Chong et al. (2009)

CSTR: continuous stirred tank reactor; UASB: up-flow anaerobic sludge blanket reactor; COD: chemical oxygen demand; POME: palm oil mill effluent

**Table 4:** Dark fermentative biohydrogen production from POME using mixed culture

Inoculum	Inoculum pre-treatment	Reactor type	Operating conditions		% H_2_	H_2_ yield	H_2_ production rate	Dominant microbes	Technique for microbial community analysis	References
			Temperature, *T*	pH						
POME anaerobic sludge	HT, 80 °C, 50 min	UASFF	37 °C	Initial = 5.2–5.8, Operation = 5.2–5.8	71.37	800 mL H_2_/g COD_consumed_	4.1 L H_2_/L day	*Clostridium *sensu stricto* 1* and *Lactobacillus*	Amplicon sequencing—Illumina MiSeq	Akhbari et al. (2021)
Anaerobic sludge from methane-producing anaerobic digester	HT, 90 °C, 60 min	CSTR	30 °C	Initial = 5.5, Operation = 5.5	30—34	249 mL H_2_/ g COD	22.22 mL H_2_/L h	*Clostridia*	Amplicon sequencing -Illumina MiSeq	Audu et al. (2021)
Thermophilic biohydrogen-producing sludge	HT, 80 °C, 60 min	Batch	55 °C	Initial = 6.0, Operation = uncontrolled	38.77	794.85 mL H_2_/L POME or 1.88 mol H_2_/mol_sugar_	ND	ND	ND	Abdullah et al. (2020)
Sugarcane cultivation soil	ND	Hungate tube	30 °C	Initial = 7, Operation = uncontrolled	ND	1617 mL H_2_/L medium	ND	*Sporolactobacillus* and *Clostridium*	Amplicon sequencing -Illumina MiSeq	Rosa et al. (2020)
Vinasse pond	ND	Hungate tube	37 °C	Initial = 7, Operation = uncontrolled	ND	1550 mL H_2_/L medium	ND	*Clostridium* and *Ruminococcus*	Amplicon sequencing -Illumina MiSeq	Rosa et al. (2020)
POME sludge	HT, 80 °C, 60 min	FBR	60 °C	Initial = 6.0, Operation = 6.0	ND	1.24 mol H_2_/mol sugar_consumed_	5.2 mmol H_2_/L h	*Thermoanaerobacterium thermosaccharolyticum* sp.	PCR-DGGE	Jamali et al. (2019)
POME anaerobic sludge	ND	Serum bottles	55 °C	Initial = 6.5, Operation = uncontrolled	ND	71 mL H_2_/g COD	7.6 mL H_2_/g COD day	*Themoanaerobacterium* sp., *T. thermosaccharolyticum, T. aciditolerans. T. brockii, Clostridium* increased overtime	PCR-DGGE	Khongkliang et al. (2019)
POME sludge	HT	ASBR	55 °C and 37 °C	Initial = 6.0, Operation = uncontrolled	ND	2.52 mol H_2_/mol sugar	10.34 mmol H_2_/L h	*Thermoanaerobacterium* sp.	PCR-DGGE	Maaroff et al. (2019)
POME digested sludge	HT, 80 °C, 60 min	UASB	55 °C	Initial = 5.2, Operation = 5.2	52	2.45 mol H_2_/mol sugar_consumed_	11.75 L H_2_/ L POME day	*Clostridium celerecrescens*, *Clostridium* sp. and Proteobacteria	PCR-DGGE	Mahmod et al. (2019)
Juice processing wastewater anaerobic sludge	HT, 105 °C, 30 min	Batch	55 °C	Initial = 6.0, Operation = uncontrolled	23.7	77 mL H_2_/g COD_removed_	ND	ND	ND	Tanikkul et al. (2019a)
Juice processing wastewater anaerobic sludge	HT, 105 °C, 30 min	Batch	37 °C	Initial = 6.0, Operation = uncontrolled	31	182 mL H_2_/g COD or 7.96 mmol/g COD	23.37 mL H_2_/ h	ND	ND	Tanikkul et al. (2019b)
POME anaerobic sludge	HT, 90 °C, 60 min	UASFF	37 °C	Initial = 5.0–5.2, Operation = uncontrolled	57.11	1021.74 mL H_2_/g COD_consumed_	5.29 L H_2_/ L day	ND	ND	Zainal et al. (2019)
POME anaerobic sludge	HT, 100 °C, 60 min	Serum bottles	50 °C	Initial = 5.5, Operation = uncontrolled	ND	28.47 mL H_2_/g COD_consumed_	ND	ND	ND	Zainal et al. (2018)
Sewage anaerobic sludge	HT, 100 °C, 60 min	Batch	35 °C	Initial = 6.5, Operation = uncontrolled	56.65	2.58 mmol H_2_/g COD or 135.79 mL H_2_/L POME	11.32 mL H_2_/L POME h	ND	ND	Garritano et al. (2017)
POME sludge	HT, 80 °C, 60 min	ASBR	60 °C	Initial = 6.0, Operation = 6.0	ND	1.6 mol H_2_/mol sugar	61.5 mmol H_2_/L day	*Thermoanaerobacterium thermosaccharolyticum*	16S rRNA Identification	Jamali et al. (2017)
POME digested sludge	HT, 80 °C, 60 min	Serum bottles	60 °C	Initial = 5.8, Operation = uncontrolled	ND	1.24 mol H_2_/mol glucose	0.181 mmol H_2_/L h	ND	ND	Mahmod et al. (2017)
POME digested sludge	HT, 100 °C, 60 min	UASFF	38 °C	Initial = 5.5, Operation = uncontrolled	56.6	ND	0.514 L H_2_/g VSS	ND	ND	Mohammadi et al. (2014)
Immobilised POME sludge	HT, 80 °C, 50 min	UASB	37 °C	Initial = 5.5, Operation = 5.5	37.1	ND	0.589 L H_2_/ L POME h	ND	ND	Singh et al. (2013a)
POME anaerobic sludge	HT, 85 °C, 60 min	ASBR	37 °C	ND	50	940 mL H_2_/g COD_consumed_	6.7 L H_2_/ L day	*Streptococcus macedonicus*, *Lactobacillus agilis* and *Clostridium butyricum* CGS6	Conventional cultivation – 16S rDNA Identification	Badiei et al. (2012)
Anaerobic sludge	HT, 105 °C, 90 min	Serum bottles	44 °C	Initial = 7.0, Operation = uncontrolled	ND	0.68 mmol H_2_/g COD	ND	*Clostridium* spp. and *Thermoanaerobacterium* spp.	qPCR	Leaño et al. (2012)
*Thermoanaerobacterium*-rich sludge	ND	CSTR	60 °C	Initial = 5.5, Operation = uncontrolled	ND	4.2 L H_2_/L POME	ND	*T. thermosaccharolyticum, T. aciditolerans*	PCR-DGGE	Mamimin et al. (2012)
POME digested sludge	HT, 85 °C, 20 min	Batch	36 °C	Initial = 5.8, Operation = 5.8	ND	1.32 L H_2_/L POME	0.144 L H_2_/ L h	ND	ND	Rasdi et al. (2012)
POME anaerobic sludge	HT, 90–95 °C, 30 min	Serum bottles	37 °C	Initial = 6.0, Operation = uncontrolled	ND	27.09 mL H_2_/g COD	41.91 mL H_2_/L h	*Clostridium paraputrificum*, *Weissella soli*, *C. butyricum*, *C. hydrogeniformans*, *C. beijerinckii* and *Clostridium* spp.	PCR-DGGE	Yossan et al. (2012)
POME anaerobic sludge	HT, 90–95 °C, 30 min	Serum bottles	55 °C	Initial = 6.0, Operation = uncontrolled	ND	26.63 mL H_2_/g COD	49.34 mL H_2_/L h	*C. paraputrificum*, *C. butyricum*, *Thermoanaerobacterium thermosaccharolyticum*, *C. baratii* and *Clostridium* spp.	PCR-DGGE	Yossan et al. (2012)

POME: palm oil mill effluent; HT: heat treatment; ND: no data; ASBR: anaerobic sequencing batch reactor; CSTR: continuous stirred tank reactor; FBR: fluidised bed reactor; UASB: up-flow anaerobic sludge blanket reactor; UASFF: up-flow anaerobic sludge blanket fixed-film reactor; COD: chemical oxygen demand; PCR-DGGE: polymerase chain reaction denaturing gradient gel electrophoresis; qPCR: quantitative polymerase chain reaction

Different *Clostridium* species exhibit different metabolic activities, and their relative abundance vary depending on the operational conditions. Yossan et al. (2012) found that *C. paraputrificum* is the dominant member of the biohydrogen-producing community under all temperatures. In this study, *C. butyricum* was detected in the biohydrogen reactor operated at 37–55 °C, whereas *C. beijerinckii* and *C. hydrogeniformans* were only present at 37 °C. Biohydrogen production reactor at thermophilic condition was dominated by *C. thermopalmarium*, a non-cellulolytic biohydrogen-producing bacteria (Yossan et al. 2012). In another study, *C. *sensu stricto contributed 800 mL H_2_/g COD_consumed_ of biohydrogen yield when treating POME with anaerobic sludge in up-flow anaerobic sludge blanket fixed-film (UASFF) reactor operated at 37 °C with the total abundance of 69.55% in the system (Akhbari et al. 2021). *C. celerecrescens* was the dominant biohydrogen producer in up-flow anaerobic sludge blanket (UASB) reactor using POME substrate operated under thermophilic condition (Mahmod et al. 2019). *Clostridia* can also be the main biohydrogen producers even though they do not dominate the whole community. Badiei et al. (2012) performed microbial community analysis on the anaerobic sludge of an anaerobic sequencing batch reactor (ASBR) operating under mesophilic temperature. 940 mL H_2_/g COD_removed_ of biohydrogen was obtained in this system. Only 20% of the relative microbial abundance were represented by *Clostridia*. The community was dominated by *Streptococcus* (50% relative abundance) and *Lactobacillus* (30% relative abundance), and the biohydrogen yield was comparable with the yield obtained by Akhbari et al. (2021) in a reactor dominated by *Clostridium*. This suggests that *Clostridia* does not have to dominate the system in order to obtain a high biohydrogen production yield. Deep metagenomics sequencing can help reveal the syntrophic relationship that may exist between *Clostridia* and the other genera not known to be the biohydrogen producers, and the connections between the different communities at different trophic levels in the reactor.

## Tools for biohydrogen microbiome analysis

Biohydrogen production through dark fermentation from organic wastes, including POME, is a complex biochemical process, carried out by microbial communities with a range of relationships between them. Dark fermentation can be divided into four key stages which are hydrolysis, acidogenesis, acetogenesis and methanogenesis (section "Microbiomes in dark fermentative biohydrogen production"). Methanogenesis is often suppressed and undesired in biohydrogen production. These processes occur synergistically in a successive manner and each stage is facilitated by a distinct guild of microorganisms. A robust and efficient dark fermentation system requires a delicate balance of microbial population dynamics and metabolic activities among different guilds or trophic groups of the biohydrogen-producing microbiomes. Understanding of the microbial ecology of the dark fermentation process can help to improve the performance towards maximising biohydrogen production, and ensure that this process is economically feasible.

A range of techniques have been used in characterising the complex biohydrogen-producing microbial communities, from conventional cultivation-dependent approaches to cultivation-independent approaches. The advanced multi-omics technologies are also increasingly being use for this purpose. Cultivation-dependent method have contributed in the discovery of many key microbial species in biohydrogen-producing bioreactors from organic industrial waste and POME (Alvarado-Cuevas et al. 2015; Harun et al. 2012; Hsieh et al. 2016; Mishra et al. 2017; Noparat et al. 2012; Singh et al. 2014; Yin and Wang 2017; Zhang et al. 2015). While economical and a generally useful method to shed light on some key members, not many can be characterised this way, particularly when a system-based approach is required. Some key taxa also have their syntrophic partners belonging to different functional guilds (Lim et al. 2020). This method is further limited by species-specific morphological variations since some microorganisms share similar morphological, physiological or biochemical characteristics which makes the classification challenging (Lim et al. 2020). While cultivation method might be time consuming and labour intensive, it is the only technique to characterise a specific strain in detail. Current-omics technologies also require more reference genomes to evaluate the biohydrogen-producing microbiomes sequence data. Therefore, culture-dependent method will remain essential for studying the microbial diversity in biohydrogen-producing microbiomes. Recently, novel biohydrogen-producing bacteria, *Clostridium sartagoforme* NASGE 01 and *Enterobacter cloacae* NASGE 02 were isolated from sago industrial effluent using this method (Nizzy et al. 2020).

Advancement in molecular biology and DNA sequencing techniques has enabled various culture-independent methods to be used to study the microbiomes in biohydrogen-producing reactors. Denaturing gradient gel electrophoresis (DGGE) and single-strand conformation polymorphism (SSCP) are among the microbiome fingerprinting techniques used to evaluate and compare different microbiomes in dark fermentation from organic wastes and POME. Both techniques involve polymerase chain reaction (PCR) amplification of a hypervariable region of the 16S rRNA gene and migration of the PCR product fragments on polyacrylamide gel that will provide different banding patterns, which reflect the structure of microbial communities and species abundance. Using PCR-DGGE, the genus *Megasphaera* sp. was identified as the main biohydrogen producer with 14% relative abundance, in thermophilic dark fermentation reactor of sugarcane stillage inoculated with granular sludge of a sugarcane stillage treatment plant. *Clostridia* were not detected in this system (Santos et al. 2014). While in dark fermentative biohydrogen production of beer lees inoculated with non-pre-treated garbage compost, using PCR-DGGE, *C*. *roseum* was found to be the prevalent biohydrogen producers in all high biohydrogen-producing batch fermentations, whereas *C. perfringens* and *C. sporogenes* were detected in low biohydrogen-producing batch fermentations (Bando et al. 2013). The presence of *Bifidobacterium* spp. and *Lactobacillus* spp. inhibited biohydrogen production through substrate competition with biohydrogen producers. Biohydrogen-producing species such as *C. butyricum* and *C. tyrobutyricum* were also found as the substrate competitors in biohydrogen fermenter dominated by *C. pasteurianum* (Lin et al. 2011). When POME was used as substrate, PCR-DGGE is still among the commonly used methods in studying the biohydrogen-producing microbiomes. The genus *Thermoanaerobacterium,* such as *T. thermosaccharolyticum,* was often reported as the main biohydrogen producers in thermophilic POME dark fermentation using POME anaerobic sludge as inoculum source (Jamali et al. 2019; Khongkliang et al. 2019; Maaroff et al. 2019).

The use of SSCP to investigate biohydrogen-producing microbial community structure is still limited. Using SSCP, operational pH of a continuous stirred tank reactor (CSTR) fed with glycerol was found to change the structure of the dominant microbial populations (Silva-Illanes et al. 2017). Hydraulic retention time (HRT) changed the metabolic pattern and the composition of subdominant microorganisms such as *Enterococcus*, *Prevotella*, *Sutterella*, *Pseudomonas* and *Acinetobacter,* ultimately affecting the ability of the consortium to produce biohydrogen. In general, DGGE and SSCP are not quantitative, more labour intensive, time consuming, prone to PCR biases and has low resolution in complex microbiome profiles (Kumar et al. 2018). Nevertheless, these microbiome fingerprinting techniques could remain useful for quick screening purposes, and to acquire a glimpse of biohydrogen-producing microbiomes from a large number of samples.

Quantitative PCR (qPCR) has also been used in studying several biohydrogen reactors using organic wastes (including POME) as substrates, to quantify the changes of specific microbial populations (Lay et al. 2010; Leaño et al. 2012; Pugazhendhi et al. 2017). In contrast to the common PCR which is qualitative, qPCR can accurately quantify the copy number of genes of interest in a sample by measuring the fluorescence of a specific probe used for amplification (Lim et al. 2020; Tolvanen and Karp 2011). This technique eliminates post-PCR target analysis, cheaper and offers a fast, accurate and simple approach for high-throughput analysis (Nurmi et al. 2002). Individual taxa or guilds in biohydrogen microbiomes can also be quantified using fluorescent in situ hybridisation (FISH) technique. In FISH, cells of interest is hybridised with a specific fluorogenic oligonucleotide probes and its relative abundance is then measured by quantifying the ratio of the hybridised cells to the total cell count using a fluorescence microscope. FISH probes Tbm1282, Ccs432 and Tbmthsac184 specific for detection of *Thermoanaerobacterium*, *Caldicellulosiruptor* and *T. thermosaccharolyticum* have been designed and used to assess the microbial composition in thermophilic and extreme thermophilic biohydrogen-producing reactors fed with POME, lignocellulosic hydrolysate and synthetic sugars (O-Thong et al. 2008). FISH overcomes the limitations of PCR-based molecular techniques. Nevertheless, cell hybridisation is time consuming, making FISH less suitable for high-throughput community structure investigation (Ravenschlag et al. 2001). Detection of novel microorganisms may also be challenging, the probe design and selection require some information on the community structure prior to the analysis (Lim et al. 2020).

High throughput next generation -omics technologies are increasingly being employed to better understand the complex microbiomes driving dark fermentative biohydrogen production. Amplicon sequencing, metagenomics and metaproteomics have all been employed in this context. Amplicon sequencing also known as metaprofiling is a culture-independent technique to profile the taxonomic diversity, structure and composition of a microbiome based on a marker gene (Escobar-Zepeda et al. 2015). 16S rRNA genes have been exclusively used as a marker gene for library preparation through PCR amplification in studies of microbial communities, including biogas-producing microbiomes (Sharpton 2014; Tonge et al. 2014). Using amplicon sequencing method, biohydrogen production using sake lees was found to be enhanced when the microbial community in the system changed from *Bacillus muralis* and *B. cereus* as the dominant taxa*,* to *Pantoea agglomerans*, *C. acetobutylicum* and *C. butyricum* (Choiron et al. 2020). Besides, with amplicon sequencing, *Sporolactobacillus* was the dominant taxa with relative abundance 97% in the fermentation of POME using microbial consortia from sugarcane cultivation soil (Rosa et al. 2020). *Sporolactobacillus* is an anaerobic facultative bacterium producing lactic acid. Although its role in biohydrogen production is unknown, its metabolic by-products could be used as substrates for biohydrogen production by other microorganisms. Amplicon sequencing is commonly done on an Illumina MiSeq platform (Akhbari et al. 2021; Audu et al. 2021; Martinez-Burgos et al. 2020; Yang and Wang 2019) while Ion Torrent platform has also been used in several studies (Cho et al. 2018; Oliveira et al. 2020). A few studies have also attempted to predict the community functions from amplicon sequencing of biohydrogen microbiomes using bioinformatic tools, such as PICRUSt (Li et al. 2020; Yin and Wang 2021) mostly using other organic wastes. So far, this has not been reported for POME. Amplicon sequencing is the best and economical option to understand the microbial community members in general, but it has limitations and may result in biases (Lim et al. 2020), leading to the increasing applications of shotgun metagenomics.

Shotgun metagenomics independently sequences total genomic DNA retrieved directly from a sample to produce reads that align to various genomic locations for the countless genomes present, including the non-microbes (Sharpton 2014). Metagenomic tools could unravel the vast taxonomic diversity, metabolic function potential and physiology of uncultivated microorganisms, including the novel and rare taxa, and previously unknown metabolic pathways (Vanwonterghem et al. 2014). A few studies have investigated the biohydrogen-producing microbiomes using metagenomics (Mazareli et al. 2020; Soares et al. 2018; Villa Montoya et al. 2020). Mazareli et al. (2020) used metagenomics to correlate taxonomic diversity of indigenous microbial biomass with the performance of biohydrogen reactor fed with banana wastes under mesophilic temperature. Using FMAP (Functional Mapping and Analysis Pipeline) for metagenomic and metatranscriptomic studies tool, *Clostridium* and *Lactobacillus* were the dominant indigenous acidogenic bacteria, and the main genes encoding key enzymes involved in the fermentation were found to be related to carbohydrate metabolism, acidogenesis and biohydrogen production enzymes such as glucose-6-phosphate dehydrogenase, fructokinase, lactate dehydrogenase and pyruvate ferredoxin oxidoreductase. Metagenomic study by Villa Montoya et al. (2020) reported domain Bacteria represented 97.2% relative abundance with the predominance of genera *Clostridium* (87.9% relative abundance) in the mesophilic biohydrogen-producing bioreactor fed with coffee wastes. Gene identifications showed that 8.3% of the genes were corresponded to anaerobic degradation enzymes mainly for the production of organic acids and alcohols and may be associated with the metabolic potential of *Clostridium* sp. In addition, 37 KEGG orthologues (KOs) were identified to be associated with biohydrogen production, highlighting enzymes pyruvate-ferredoxin oxidoreductase, anaerobic carbon-monoxide dehydrogenase, formate dehydrogenase and ferredoxin hydrogenase. Genes related to these enzymes were mainly found in *Clostridium* sp. (Villa Montoya et al. 2020).

Breakthroughs in next generation sequencing (NGS) technologies has also led to another subfield of -omic technologies, which is metaproteomics. Metaproteomics profiles enzymes and proteins in microbiomes, and can potentially link the function of a protein to a taxon and its metabolic activities (Chistoserdova 2009; Lim et al. 2020). Metaproteomics has been widely applied in studying anaerobic digester bioreactors and human gut microbiome, but its application is still limited in investigating the microbiomes of biohydrogen dark fermentation. Previously, metaproteomics was used to establish the relationship between phylogeny, function, and metabolic activity of biohydrogen and methane co-production microbiomes from food waste (Jia et al. 2017). A total of 651 bacterial proteins and 477 archaeal proteins were detected in the study, revealing the complexity and metabolic diversity during the biogas production process. The study also revealed that the key bacterial proteins from *Gammaproteobacteria*, *Clostridia* and *Bacilli* related to biohydrogen production came from pyruvic acid decarboxylase and formic acid decomposition pathway in carbohydrate metabolisms.

## Future outlook

Biogas (i.e. methane) microbiomes are more widely and intensively studied than biohydrogen, despite the fact that these two processes share many biochemical and metabolic routes. This is probably due the more advanced research and wider adoption of anaerobic digestion for biogas production as cleaner energy production technology in the society. It has been demonstrated that methane production is directly linked to the composition of the anaerobic digester microbiomes, in addition to the microbial metabolism, which is dependent on the environmental parameters of the reactor (Campanaro et al. 2020). This makes understanding of the microbial composition of a bioreactor and their behaviour a critical aspect in the quest for a feasible biohydrogen production via dark fermentation. Pugazhendhi et al. (2019) reviewed the microbiomes involved in the anaerobic hydrogen-producing granules (HPG). Granulation increases the reaction efficiency of a fermenter, compared to using sludge. The dominant taxa in the microbial community of reactor systems employing HPG has been discussed, allowing the monitoring of the microbial species for easier control of the kinetic parameters, and contributes to the development of stable bioprocess system (Pugazhendhi et al. 2019). This suggests the importance of meta-analysis of hydrogen-producing microbial community from different reactor systems, and the correlation with their physicochemical parameters and reactor performance.

A summary of the molecular tools in analysing the biohydrogen-producing microbial community has recently been published (Kumar et al. 2020), describing the “targeted” molecular tools (e.g. FISH, qPCR) and the advantages of NGS in providing quicker and more comprehensive investigation. A combination of culture-dependent approach, targeted molecular tools and NGS, and multi-omics are definitely the way forward in providing a system-based understanding of the biohydrogen microbiomes. Multi-omics of this engineered reactor system can also benefit from the rapidly expanding experimental and computational tools for investigating human and environmental microbiomes, allowing for deeper understanding of the community structure and functions from the -omics data. This includes the advancements in co-occurrence network, genome-scale metabolic model, protein–protein interaction network, the metabolic-driven metabolomics network (Liu et al. 2020), and the integration of all the -omics data. This is in addition to the need for best practices for analysing the microbiomes towards a unified approach in the analysis of reactor systems.

Knowledge obtained from the -omics techniques can be used to engineer a desired community structure, towards maximising productivity of an engineered system, and balancing the effects of any perturbations. Tools for manipulating community structure in situ are also being investigated. CRISPR/Cas-related system has been used in a targeted genome editing of specific microorganisms within a complex microbial community (Rubin et al. 2020), paving the way for manipulation of microbiomes in many different applications, possibly in the biogas and biohydrogen-producing reactor system too. There is still a long way to go before this precise gene and genome manipulation system can be applied in a complex community like the anaerobic digester’s, but it is important to first have the full understanding of the microbial community and the relationships with the physicochemical parameters in controlling the production yield and rate.

## Conclusion

Biohydrogen is a common by-product of many bacterial metabolic pathways during dark fermentation. Microbial communities involved in dark fermentation are phylogenetically and functionally diverse which contribute to biohydrogen production from the breakdown of complex organic substrates, such as POME and other industrial wastes. As a system which relies on microbial metabolisms, insights on the microbial members present in the reactor is important towards obtaining a robust and efficient biohydrogen production system. Numerous molecular tools for screening, quantification and identification of biohydrogen-producing microbial communities have been used to correlate the phylogeny, interspecies interactions and their function to dark fermentative biohydrogen process. Currently, DGGE and amplicon sequencing are widely used in the study of biohydrogen microbiomes. The use of -omics technologies in biohydrogen research are still relatively limited, compared to the more widely investigated anaerobic digester’s microbiomes for biomethane production. We believe similar advanced tools can be applied to biohydrogen-producing reactors too, with the prospect to unravel the limitless potential of the microbial members in the system.

## Data Availability

Not applicable.

## Abbreviations

POME: Palm oil mill effluent
rRNA: Ribosomal RNA
RDP: Ribosomal Database Project
NCBI: National Center for Biotechnology Information
MAGs: Metagenome-assembled genomes
MiDAS: Microbial Database for Activated Sludge
ASV: Amplicon sequence variant
CO_2_: Carbon dioxide
H_2_: Hydrogen
CH_4_: Methane
SCFA: Short-chain fatty acid
PFL: Pyruvate formate lyase
Fd: Ferredoxin
PFOR: Pyruvate ferredoxin oxidoreductase
NADH: Nicotinamide adenine dinucleotide
NFOR: Nicotinamide adenine dinucleotide ferredoxin oxidoreductase
ATP: Adenosine triphosphate
BOD: Biological oxygen demand
COD: Chemical oxygen demand
UASFF: Up-flow anaerobic sludge blanket fixed-film
UASB: Up-flow anaerobic sludge blanket
ASBR: Anaerobic sequencing batch reactor
DGGE: Denaturing gradient gel electrophoresis
SSCP: Single-strand conformation polymorphism
PCR: Polymerase chain reaction
CSTR: Continuous stirred tank reactor
HRT: Hydraulic retention time
qPCR: Quantitative polymerase chain reaction
FISH: Fluorescent in situ hybridisation
FMAP: Functional Mapping and Analysis Pipeline
KOs: KEGG orthologues
NGS: Next generation sequencing
HPG: Hydrogen-producing granules
